# Association between joint physical activity and alcohol intake and all-cause mortality in patients with cardiovascular disease: findings from NHANES 2007–2018

**DOI:** 10.3389/fnut.2025.1616561

**Published:** 2025-09-02

**Authors:** Shiyang Zhang, Yeji Zhuo, Chunyan Zhu, Tong Zhou, Zongtao Wang, Jianyi Zheng, Tudi Li, Rong Chen, Dong Lin, Zhixin Xie, Zhenyang Fu, Zhihuan Zeng, Kaitong Chen

**Affiliations:** Department of Cardiology, The First Affiliated Hospital of Guangdong Pharmaceutical University, Guangzhou, China

**Keywords:** cardiovascular disease, physical activity, alcohol, light drinking, NHANES

## Abstract

**Background:**

Physical activity and alcohol consumption are both highly prevalent in many countries, while the evidence related to joint effect on physical activity and alcohol consumption on patients with cardiovascular disease (CVD) was still deficient. This study was aimed to investigate the association between joint moderate-to-vigorous physical activity (MVPA) and alcohol intake and all-cause mortality in patients with CVD.

**Methods:**

A total of 4,047 participants from the National Health and Nutrition Examination Survey (NHANES) with self-reported CVD history, exercise and alcohol consumption amounts were included this study. Participants were stratified into 12 groups by MVPA level (none/insufficient/sufficient) and alcohol intake (abstainer/light/moderate/heavy). Restricted cubic spline and Cox regression models assessed mortality associations.

**Results:**

MVPA showed an L-shaped association with mortality, while alcohol exhibited a U-shaped pattern. The lowest observed mortality risk occurred in patients with MVPA ≥150 min/week and alcohol consumption 8.4–15.4 drinks/week (HR = 0.348, 95% CI: 0.169–0.667). Comparably lower risk was seen with MVPA <150 min/week plus light alcohol intake (<8.4 drinks/week) (HR = 0.400, 95% CI: 0.267–0.588).

**Conclusion:**

Among patients with established CVD, any level of MVPA was associated with survival benefits. The greatest mortality reduction occurred with >150 min/week of MVPA alongside light to moderate alcohol consumption (<15.4 drinks/week). However, comparable survival benefits were observed with <150 min/week of MVPA when coupled with light alcohol intake. MVPA remains the cornerstone for mortality risk reduction in this population.

## Graphical abstract

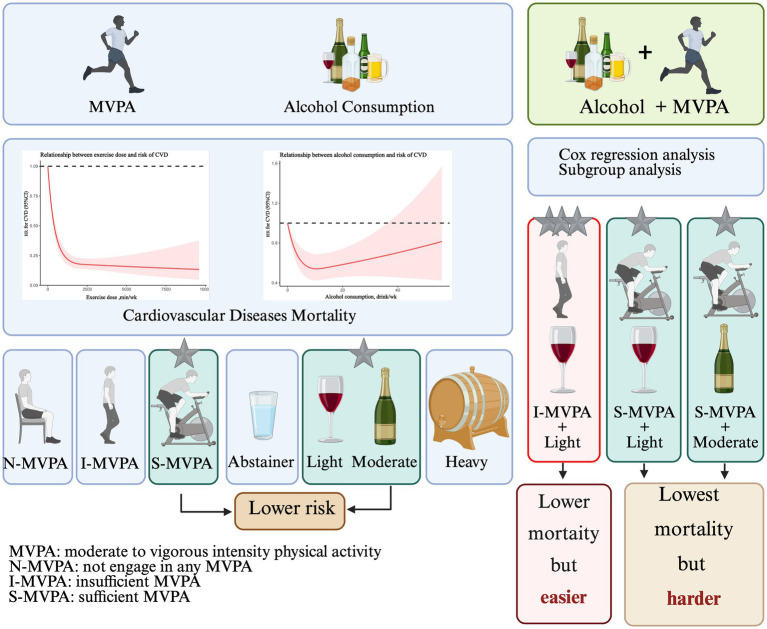

## Introduction

Cardiovascular disease (CVD) has been a leading cause of death in the world and a major obstacle to sustainable development of human society (1). Correlational research showed that there were about 422.7 million cases of CVD and 17.92 million CVD deaths in 2015 in each world region, and ischemic heart disease was the leading cause of cardiovascular health decline worldwide, followed by stroke (1). Evidence from the Global Burden of Disease (GBD) suggests that prevalent cases of total cardiovascular disease (including stroke) nearly doubled from 271 million [95% uncertainty interval (UI) 257–285] in 1990 to 523 million (497–550) in 2019 (2). Based on the latest GBD study, ischemic heart disease (IHD) had the highest global age-standardized rate of disability-adjusted life years (ASRDALYs) among all diseases, at 2275.9 per 100,000 population (3). The age-standardized prevalence rate was 3610.2 per 100,000 population, and the mortality rate was 108.8 per 100,000 population, making it the leading cause of the global noncommunicable disease burden in 2021 (4). Fortunately, the occurrence of CVD can be prevented by greatly managing those hazardous factors and early intervention (5). According to a study which aimed to forecast geospatial trends of modifiable cardiovascular risk factors from 2025 to 2050, high systolic blood pressure (SBP) would contribute to the highest age-standardized disability-adjusted life years (DALYs) in 2050, followed by high low-density lipoprotein cholesterol (LDL), high body mass index (BMI), tobacco use and high fasting plasma glucose (FPG) (6). In 2022, The American Heart Association introduced the concept of “healthy lifestyle” in relation to cardiovascular health—“Life’s Essential 8 (LE8)” as a cardiovascular health score. In LE8, there are eight lifestyle healthy behaviors, including diet, physical activity, smoking, sleep health, BMI, FPG, non-high-density lipoprotein (non-HDL) cholesterol and blood pressure (7). Among them, the exercise is the key point. Data from population-based cohort study have demonstrated the beneficial effects of exercise training on cardiovascular health, while physical inactivity has long been considered as a critical cardiovascular risk factor for the development of CVD (8, 9). Scientific research indicated that physical activity was the key factor in reduction of cardiometabolic risk and clinical terminus in CVD patients (10). Most researches had manifested that a physically active lifestyle and sedentary time reduction could be superb tactics for life-long CVD prevention, because physical activity can effectively influence CVD risk factors such as BMI, hypertension, diabetes, and blood lipids (5). World health organization (WHO) recommended that at least 150 min moderate intensity exercise or 75 min vigorous intensity exercise per week could be preventive on CVD risk (11).

In addition to physical activity, alcohol consumption is a widely studied behavioral factor in cardiovascular health. The impacts of ethanol on cardiovascular system depend on the amount of alcohol consumed and the pattern of drinking. In many prospective studies moderate intake (about one to two drinks per day, or 100 g of alcohol per week) is associated with somewhat lower incidence of stroke and myocardial infarction than no alcohol intake (12, 13). Previous studies had proved a J-shaped curve that light to moderate drinkers have less risk of CVD than abstainers, and heavy drinkers are at the highest CVD risk (14). But a genetic epidemiology study reported that alcohol consumption of all amounts was associated with increased CVD risk (15). Contrast results appeared among the studies on the association between ethanol use and CVD risk made the cardiovascular effects of alcohol intake still controversial (14, 16, 17).

Physical activity and alcohol consumption are both highly prevalent in many countries. Whether CVD patients who exercise and drink alcohol has a greater mortality reduction compared to those only exercise or drink. And if opportune alcohol use does not have healthy effect for cardiovascular, whether alcohol consumption can diminish the cardiovascular protective effects of exercise. Former research focused on single effect of exercise or alcohol use on preventing the occurrence of CVD and reducing disease progress. However, evidence regarding the interact association between physical activity and alcohol consumption in patients with CVD is lacking. This study was aimed to investigate the association between joint physical activity and alcohol intake and all-cause mortality in patients with CVD.

## Methods

### Study population

**Figure 1:**
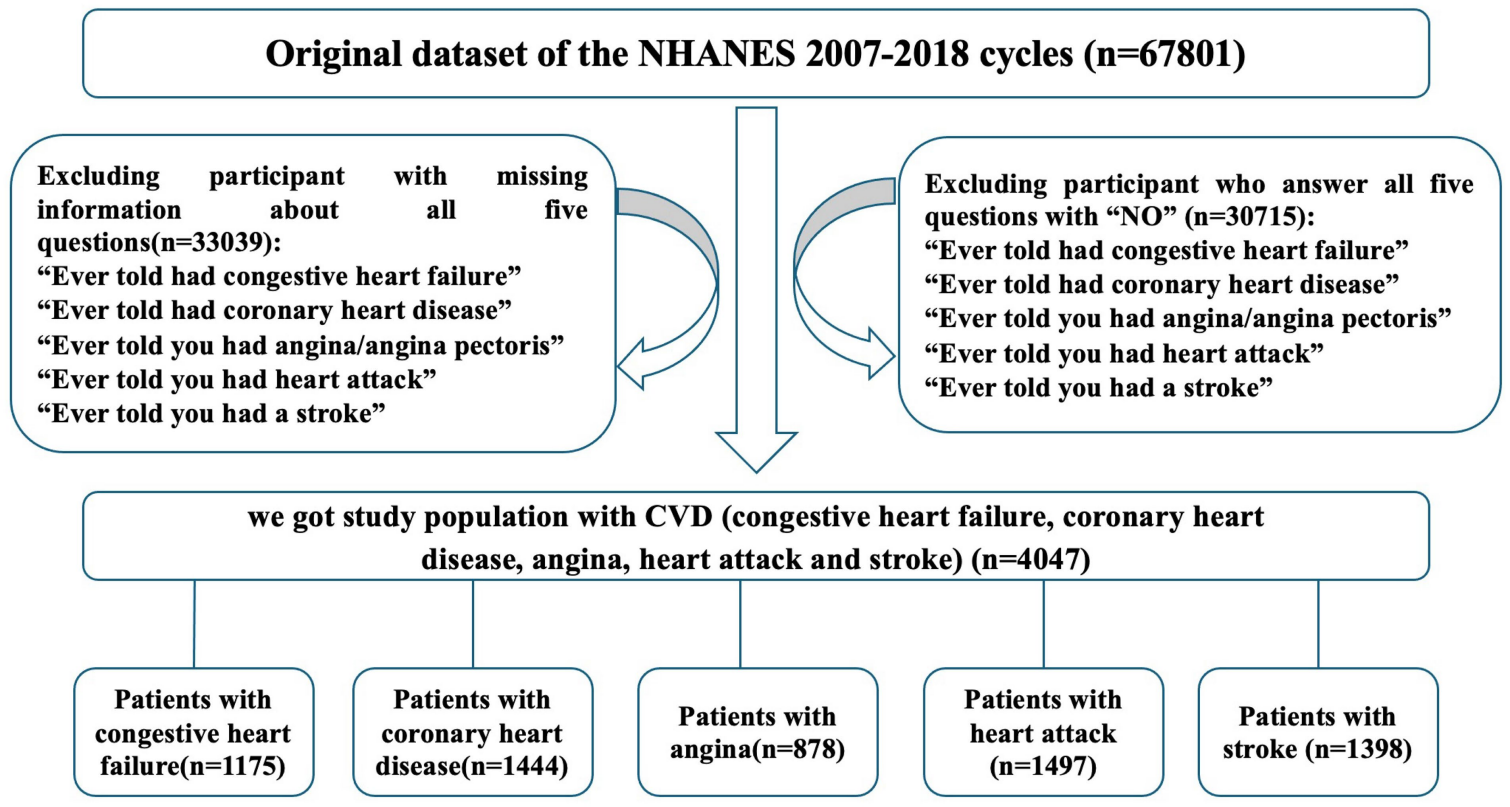
Flowchart depicting participant selection in study. A total of 67,801 adults were identified across the NHANES cycle between 2007 and 2018. Participants reported no congestive heart failure, coronary heart disease, angina, heart attack and stroke were eliminated. Meanwhile, samples with missing information were excluded. A total of 4,047 patients with CVD (congestive heart failure, coronary heart disease, angina, heart attack and stroke) were included in our study.

The National Health and Nutrition Examination Survey (NHANES) used a complex, multistage, probability sampling method to collect nationally representative health related data on the United State population. It was conducted periodically before 1999 and on a continuous basis thereafter. Data were obtained by in-person interview, mobile physical examination, and laboratory tests. In this analysis, the NHANES data from six cycles (2007–2008 through 2017–2018) were combined and merged with the mortality data described later. A total of 67,801 adults were selected from NHANES database, and 33,039 participants with missing or invalid information about CVD were excluded. Meanwhile, 30,715 people without CVD history were left out. Then 4,047 patients with self-reported CVD were involved in the analysis, including patients with congestive hearts failure (*n* = 1,175), coronary heart disease (CHD, *n* = 1,444), angina (*n* = 878), heart attack (*n* = 1,497) and stroke (*n* = 1,398). The NHANES 2007–2018 with follow-up mortality data were used to examine the associations of physical activity and alcohol use with all-cause mortality. The flowchart depicting participants selection in the study was shown in Figure 1.

### Evaluation of CVD

CVD were defined as individuals with congestive heart failure, CHD, angina, heart attack or stroke. The outcomes of CVD were obtained through questionnaire data. Five questions included “Ever told had congestive heart failure,” “Ever told had coronary heart disease,” “Ever told you had angina/angina pectoris,” “Ever told you had heart attack” and “Ever told you had a stroke” were answered by the individuals, and the participants with “Yes” were considered having the history of one of these diseases. The individuals answer all five questions with “No” were identified having no history of CVD.

### Study covariates

Information on covariates was available through baseline questionnaires, including age, gender, race/ethnicity (non-Hispanic White, non-Hispanic Black, Mexican American, and others such as other Hispanic, Asian, and multi-racial), education level [under high school (less than 11th grade include 12th grade with no diploma), high school or equivalent, some college or AA degree, college or above], household income (under $20,000 or over $20,000), marital status (living with partner or did not live with partner), occupation (employee of a private company, self-employed in own business, professional practice or farm, state government employee, local government employee, federal government employee or working without pay in family business or farm), sleeping quality (trouble sleeping, no trouble sleeping), blood pressure, HDL, LDL, triglyceride (TG), total cholesterol (TC), BMI, FPG, glycosylated hemoglobin (HbA1c) and insulin.

### Assessment of amount of physical activity

Physical activity amounts were collected through questionnaire. Moderate intensity physical activity (MPA) and vigorous intensity physical activity (VPA) was defined as “exercise which causes a moderate increase in breathing and heart rate” and “exercise that causes a high intensity increase in respiration and heart rate” (18). Total time spent in moderate to vigorous intensity physical activity (MVPA) in a typical week was calculated for each participant by adding weekly minutes of MPA and VPA across the three domains, and 1 min of VPA was counted as 2 min of MPA (19). According to the total time of MVPA recommended by WHO (11), participants were divided into three groups: “N-MVPA” for those who did not engage in any MVPA in a typical week (exercise time = 0 min), “I-MVPA” for those who engaged in insufficient (0 < exercise time < 150 min) MVPA in a typical week, and “S-MVPA” for those with sufficient (exercise time ≥150 min) MVPA in a typical week.

### Assessment of alcohol consumption

Participants were asked disclose frequency and quantity of ethanol intake, they answered the questionnaire using measuring unit of “drink.” As reported by the National Institute of Health, in the United States, a “standard drink” is defined as any beverage containing 14 g of pure alcohol (20). They need to answer, “In the past 12 months, on those days that you drank alcoholic beverages, on the average, how many drinks did you have in a week, a month or a year?.” For data from 2007 to 2016, if individuals reported alcohol consumption in the level of month, the data were divided by 4 to estimate their weekly alcohol use. Similarly, if participants reported their alcohol use in year level, the data were divided by 52. For data from 2017–2018 cycle, participants did not accurately report the frequency but a range value of alcohol consumption in the past 12 months. The average values of their alcohol consumption were then calculated.

Consistent with the approach employed by Abel et al. (21), drinking groups were defined as abstainers (0 drinks/week), light (0.1–8.4 drinks/week), moderate (8.5–15.4 drinks/week) and heavy (>15.4 drinks/week).

According to the levels of physical activity and alcohol consumption, participants were further divided into 12 groups to explore the joint effect. For example, participants with levels of N-MVPA and were divided into four groups based on their alcohol consumption level (abstainers, light, moderate and heavy). Similarly, participants with levels of I-MVPA and S-MVPA were divided into other 8 groups, respectively.

### Statistical analysis

Descriptive statistics were estimated using mean value ± standard deviation (SD) or percentage for continuous and category variables. Comparisons among groups were one-way ANOVA for continuous variables and chi-square for category variables, respectively. Outcomes were assessed within groups with paired *t* testing. The Kolmogorov–Smirnov test was used to test for normal distribution of continuous variables. Cox proportional hazards regression analysis was completed to explore independent associations of exercise and alcohol consumption with all-cause mortality in patients with CVD. The restrict cubic spline (RCS) analyses was performed to investigate the dose–response relation between exercise volume, alcohol consumption and risk of CVD. The knot quantity was 3 based on sample size considerations (*n* = 4,047) and event rates (31.3% mortality), as well as the result of Akaike information criterion (AIC) model efficacy appraisal (AIC = 4803.9). The hazards ratios (HR) and 95% CI were examined to indicate the relative risk of all-cause death by different levels of physical activity and alcohol intake, and *p*-values <0.05 were considered significant. Results were adjusted for all covariates. Analyses were performed with R software.^1^

## Results

### Baseline characteristics

A total of 4,047 samples were included in the analysis. The demography and clinical characteristics of individuals in the different physical activity groups and alcohol consumption categories are presented in Tables 1, 2.

**Table 1:** Baseline characteristics of participants in different exercise groups.

Variables	N-MVPA (*N* = 1,878)	I-MVPA (*N* = 526)	S-MVPA (*N* = 1,643)	*p*-value
Age, year	68.63 ± 12.04	67.01 ± 12.18	63.39 ± 13.47	<0.001
Gender, male	932 (49.63%)	286 (54.37%)	1,050 (63.91%)	<0.001
Race				0.011
Mexican American	192 (10.22%)	41 (7.79%)	175 (10.65%)	
Non-Hispanic White	953 (50.75%)	271 (51.52%)	842 (51.25%)	
Non-Hispanic Black	446 (23.75%)	127 (24.14%)	337 (20.51%)	
Other Hispanic	164 (8.73%)	40 (7.60%)	131 (7.97%)	
Other race	123 (6.55%)	47 (8.94%)	158 (9.62%)	
SBP, mmHg	134.61 ± 22.76	132.93 ± 21.62	130.75 ± 20.18	<0.001
DBP, mmHg	66.50 ± 15.52	67.67 ± 15.02	68.98 ± 14.38	<0.001
BMI, kg/m^2^	30.74 ± 7.60	31.08 ± 7.91	29.96 ± 6.90	0.001
HDL, mmol/L	1.28 ± 0.41	1.30 ± 0.39	1.31 ± 0.43	0.107
LDL, mmol/L	2.58 ± 0.99	2.54 ± 0.98	2.61 ± 0.93	0.697
Triglyceride, mmol/L	1.58 ± 1.13	1.81 ± 2.39	1.46 ± 1.03	0.001
Cholesterol, mmol/L	4.64 ± 1.18	4.63 ± 1.16	4.63 ± 1.13	0.922
FPG, mmol/L	7.11 ± 2.63	6.87 ± 2.87	6.68 ± 2.20	0.034
HbA1c, mmol/L	6.39 ± 1.40	6.22 ± 1.19	6.16 ± 1.24	<0.001
Insulin, pmol/L	101.58 ± 97.06	110.88 ± 115.20	93.99 ± 119.63	0.317
Income				<0.001
Under $20,000	656 (34.9%)	178 (33.8%)	472 (28.7%)	
Over $20,000	1,082 (57.6%)	323 (61.4%)	1,071 (65.2%)	
Job				0.878
Employee of private company	124 (62.94%)	51 (61.45%)	317 (64.83%)	
Self-employed in own business, farm	37 (18.78%)	15 (18.07%)	93 (19.02%)	
State government employee	9 (4.57%)	6 (7.23%)	27 (5.52%)	
Local government employee	16 (8.12%)	8 (9.64%)	36 (7.36%)	
Federal government employee	5 (2.54%)	3 (3.61%)	10 (2.04%)	
Working in family business or farm	5 (2.54%)	0 (0.00%)	4 (0.82%)	
Sleep				<0.001
Trouble sleeping	295 (15.71%)	77 (14.64%)	208 (12.66%)	
No trouble sleeping	937 (49.89%)	260 (49.43%)	741 (45.10%)	
Marital				<0.001
Live with partner	890 (47.4%)	267 (50.8%)	971 (59.1%)	
Did not live with partner	986 (52.5%)	259 (49.2%)	671 (40.8%)	
Education level				<0.001
Under high school	779 (41.48%)	161 (30.61%)	421 (25.62%)	
High school grad/GED or equivalent	447 (23.80%)	135 (25.67%)	443 (26.96%)	
Some college or AA degree	432 (23.00%)	145 (27.57%)	468 (28.48%)	
College graduate or above	211 (11.24%)	85 (16.16%)	309 (18.81%)	

MVPA, moderate to vigorous physical activity; SBP, systolic blood pressure; DBP, diastolic blood pressure; BMI, body mass index; HDL, high density lipoprotein; LDL, low density lipoprotein; FPG, fasting plasma glucose.

**Table 2:** Baseline characteristics of participants in different alcohol consumption groups.

Variables	ABSTAINER (*N* = 2,265)	LIGHT (*N* = 1,525)	MODERATE (*N* = 134)	HEAVY (*N* = 123)	*p*-value
Age, year	68.55 ± 11.60	63.90 ± 13.78	63.02 ± 13.48	58.00 ± 14.06	<0.001
Gender, male	1,133 (50.02%)	919 (60.26%)	114 (85.07%)	102 (82.93%)	<0.001
Race					0.008
Mexican American	247 (10.91%)	136 (8.92%)	9 (6.72%)	16 (13.01%)	
Non-Hispanic White	1,099 (48.52%)	827 (54.23%)	79 (58.96%)	61 (49.59%)	
Non-Hispanic Black	520 (22.96%)	326 (21.38%)	31 (23.13%)	33 (26.83%)	
Other Hispanic	190 (8.39%)	129 (8.46%)	9 (6.72%)	7 (5.69%)	
Other race-including multi-racial	209 (9.23%)	107 (7.02%)	6 (4.48%)	6 (4.88%)	
SBP, mmHg	134.46 ± 22.5	130.56 ± 20.37	134.12 ± 21.00	131.06 ± 19.83	<0.001
DBP, mmHg	66.36 ± 15.50	68.67 ± 13.95	70.72 ± 17.04	73.83 ± 15.05	<0.001
BMI, kg/m^2^	30.38 ± 7.32	30.74 ± 7.46	29.55 ± 6.84	29.24 ± 7.35	0.051
HDL, mmol/L	1.26 ± 0.39	1.31 ± 0.42	1.38 ± 0.44	1.56 ± 0.64	<0.001
LDL, mmol/L	2.58 ± 1.01	2.61 ± 0.93	2.45 ± 0.79	2.72 ± 0.79	0.399
Triglyceride, mmol/L	1.56 ± 1.32	1.53 ± 1.13	1.67 ± 1.46	1.77 ± 2.62	0.473
Total cholesterol, mmol/L	4.59 ± 1.18	4.67 ± 1.13	4.55 ± 1.08	4.97 ± 1.10	0.003
FPG, mmol/L	7.07 ± 2.62	6.74 ± 2.38	6.51 ± 1.38	6.43 ± 2.69	0.081
HbA1c, mmol/L	6.36 ± 1.33	6.21 ± 1.28	5.98 ± 1.25	5.96 ± 1.34	<0.001
Insulin, pmol/L	106.99 ± 131.88	95.41 ± 80.35	80.68 ± 59.44	62.10 ± 50.70	0.040
Income					<0.001
Under $20,000	811 (35.8%)	413 (27.1%)	39 (29.1%)	43 (35.0%)	
Over $20,000	1,276 (56.3%)	1,038 (68.1%)	89 (66.4%)	73 (59.3%)	
Job					0.244
An employee of private company	189 (63.21%)	247 (63.82%)	31 (65.96%)	25 (69.44%)	
Self-employed in own business, farm	58 (19.40%)	69 (17.83%)	13 (27.66%)	5 (13.89%)	
A state government employee	22 (7.36%)	14 (3.62%)	2 (4.26%)	4 (11.11%)	
A local government employee	17 (5.69%)	41 (10.59%)	1 (2.13%)	1 (2.78%)	
A federal government employee	9 (3.01%)	8 (2.07%)	0 (0.00%)	1 (2.78%)	
Working in family business or farm	3 (1.00%)	6 (1.55%)	0 (0.00%)	0 (0.00%)	
Sleep					0.101
Trouble sleeping	347 (15.32%)	197 (12.92%)	19 (14.18%)	17 (13.82%)	
No trouble sleeping	1,104 (48.74%)	709 (46.49%)	62 (46.27%)	63 (51.22%)	
Marital					<0.001
Live with partner	1,141 (50.4%)	850 (55.7%)	75 (56.0%)	62 (50.4%)	
Not live with partner	1,121 (49.5%)	675 (44.3%)	59 (44.0%)	61 (49.6%)	
Education level					<0.001
Under high school	906 (40.00%)	378 (24.79%)	29 (21.64%)	48 (39.02%)	
High school grad/GED or equivalent	588 (25.96%)	378 (24.79%)	29 (21.64%)	30 (24.39%)	
Some college or AA degree	490 (21.63%)	481 (31.54%)	44 (32.84%)	30 (24.39%)	
College graduate or above	272 (12.01%)	287 (18.82%)	32 (23.88%)	14 (11.38%)	

SBP, systolic blood pressure; DBP, diastolic blood pressure; BMI, body mass index; HDL, high density lipoprotein; LDL, low density lipoprotein; FPG, fasting plasma glucose.

Table 1 revealed significant differences among the three physical activity groups across various demographic and health-related variables. The results indicated that individuals in S-MVPA group were younger compared to those in I-MVPA and N-MVPA group (*p* < 0.001). There were also higher proportion of males and non-Hispanic White in the S-MVPA group. In terms of marital status, the S-MVPA group had a higher proportion to live with their partner. Additionally, individuals in the S-MVPA group also exhibited lower SBP, diastolic blood pressure (DBP), BMI, TG, HbA1c, FPG and insulin level (*p* < 0.001). The results suggested that there was no statistical significance in HDL, LDL, TC, insulin level and the types of job among different MVPA groups (*p* > 0.05). These findings highlighted the complex interplay between physical activity levels and various demographic and health-related factors in the studied population.

As shown in Table 2, HEAVY drinkers were younger. Higher proportion of male exhibited in the MODERATE (85.07%) and HEAVY (82.93%) groups compared to the ABSTAINER (50.02%) and LIGHT (60.26%) groups. Non-Hispanic Whites were more prevalent among light drinkers (*p* = 0.008). Higher rates of individuals who live with partner in the LIGHT (55.7%) and MODERATE (56.0%) groups compared to ABSTAINERS and HEAVY (50.4%) drinkers. SBP and DBP was lower in LIGHT and MODERATE compared to other groups (*p* < 0.001). HDL levels were lowest in the ABSTAINER group (*p* < 0.001) while MODERATE group had the lowest total cholesterol level (*p* = 0.003). Notably, HEAVY group had the lowest HbA1c, FPG and insulin levels (*p* < 0.001). There was no significant difference in sleep trouble, education level and job status among groups (*p* > 0.05).

### Independent effect of MVPA and alcohol use on all-cause mortality of CVD

**Figure 2:**
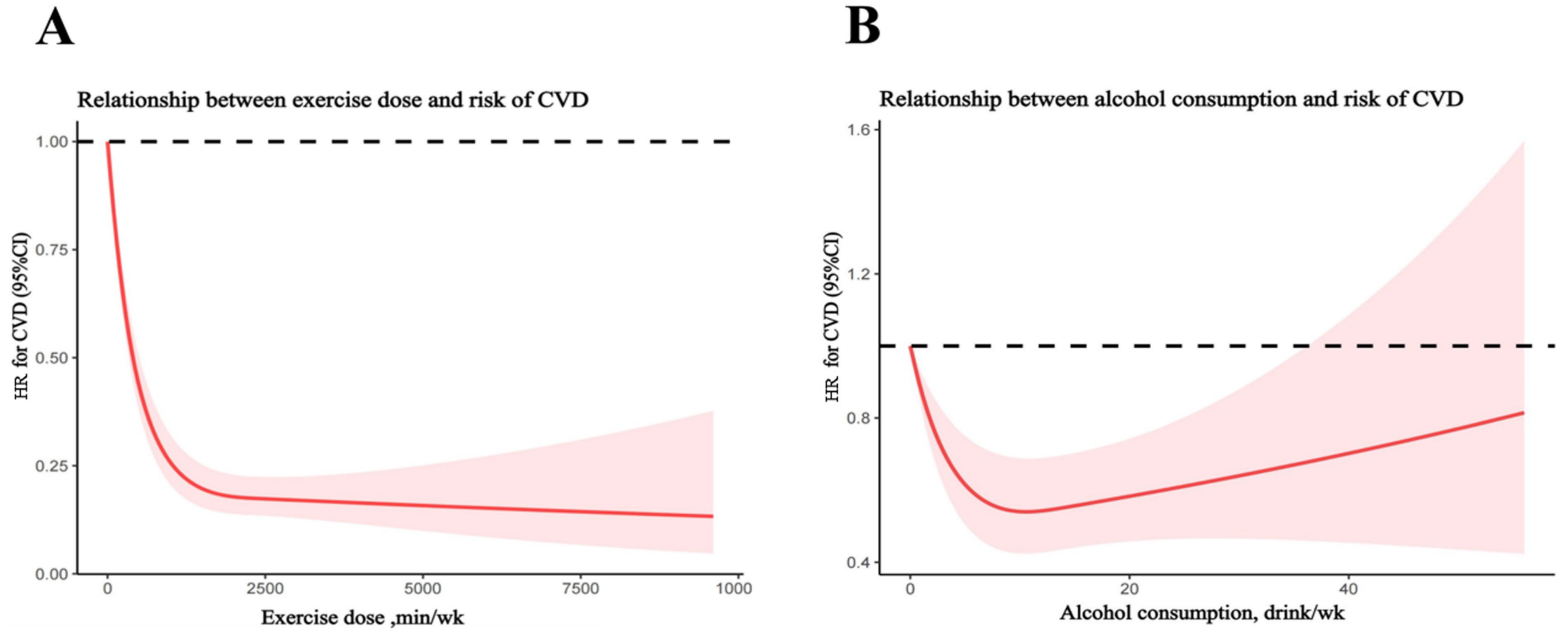
Restrict cubic spline analyses of relationship between exercise dose or alcohol consumption and risk of cardiovascular diseases (CVD). **(A)** Relationship between exercise dose and risk of CVD. **(B)** Relationship between alcohol consumption and risk of CVD.

RCS analysis indicating a curvilinear association between exercise dose and risk of all-cause mortality in patients with established CVD (Figure 2A). Any amount of exercise could be beneficial for reducing all-cause mortality, while the beneficial effect decreased very slowly as the amount of exercise continues to increase when the amount of exercise exceeded 2,000 min/week. The association between alcohol use and all-cause mortality was presented in Figure 2B. Interestingly, the model also showed that in a typical week, the risk of all-cause mortality can be reduced by increasing the amount of alcohol consumed, but when the weekly drinking amount exceeds inflection point value, the protective effect of all diseases will be gradually reduced.

**Figure 3:**
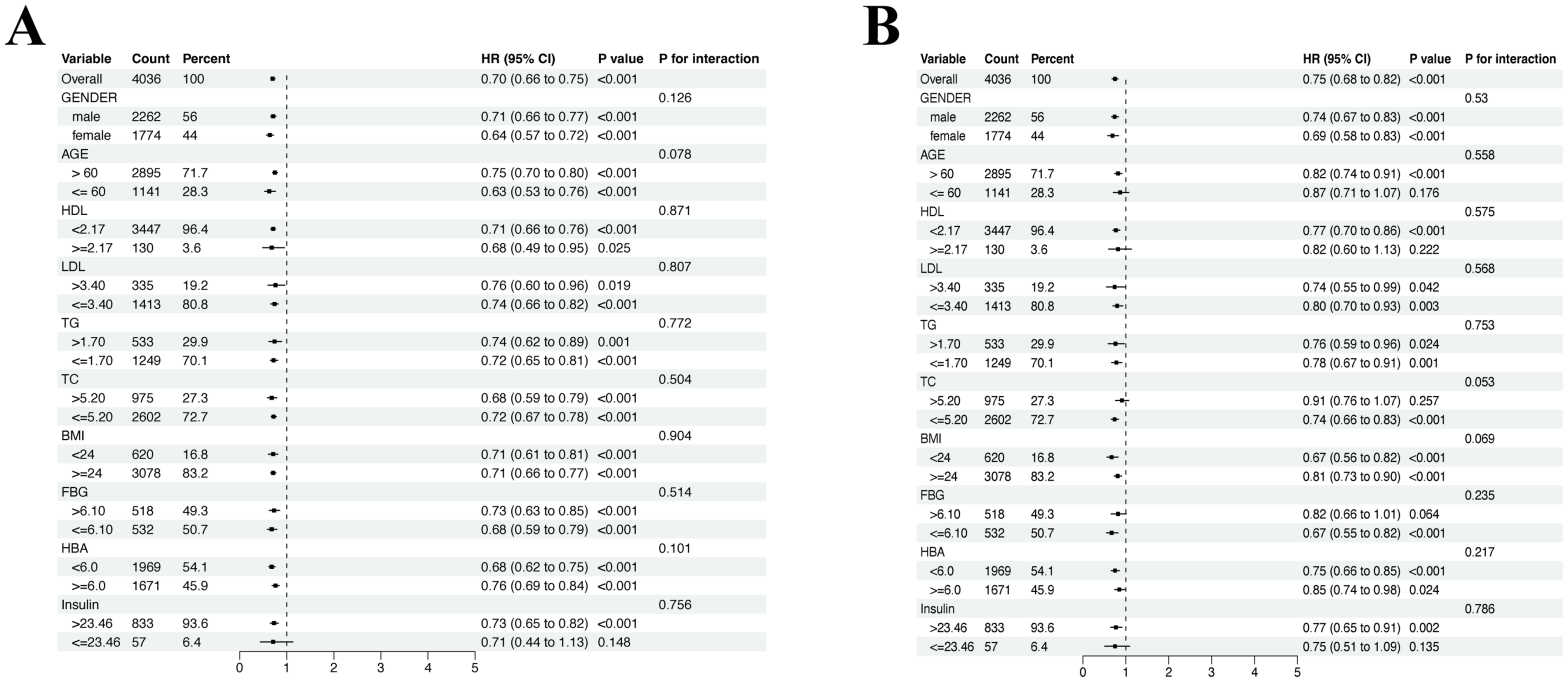
Subgroup analysis of the association between covariate groups and all-cause mortality of CVD. **(A)** Subgroup analysis in MVPA groups. **(B)** Subgroup analysis in alcohol consumption groups. Adjusted for age, gender, high-density lipoprotein (HDL), low-density lipoprotein (LDL), total cholesterol (TG), triglycerides (TC), body mass index (BMI), fasting plasma glucose (FPG), HbA1c and insulin.

Multivariate Cox analysis was used to explore the effects of covariates on all-cause mortality of CVD. As shown in Figure 3A, the protective effect of physical activity was obtained in each subgroup besides those whose insulin level less than 23.46 pmol/L. While in the subgroup analysis of alcohol consumption groups, as shown in Figure 3B, the protective effect of alcohol consumption existed especially in participants over 60 years old, HDL level less than 2.17 mmol/L, TC level less than 5.20 mmol/L, FPG level less than 6.10 mmol/L and insulin level over 23.46 pmol/L.

Cox proportional hazard regression analyses revealed that all MVPA categories were independently associated with all-cause mortality after adjusting for covariates. Lower risk of mortality exhibited in I-MVPA (HR = 0.657; 95% CI = 0.512, 0.839; *p* = 0.001) and S-MVPA (HR = 0.460; 95% CI = 0.383, 0.553; *p* < 0.001) group when compared to N-MVPA group. Surprisingly, among the alcohol consumption groups, lower hazard of mortality was also found in LIGHT (HR = 0.676; 95% CI = 0.567, 0.805; *p* < 0.001) and MODERATE (HR = 0.593; 95% CI = 0.363, 0.944; *p* = 0.032) group comparing with the ABSTAINER group except for the HEAVY group (Table 3).

**Table 3:** Independent associations of MVPA and alcohol intake with all-cause mortality among the US adults with a history of CVD.

Groups	HR^a^	95% CI	*p*-value
Physical exercise			
N-MVPA	Reference		
I-MVPA	0.657	(0.512, 0.839)	0.001
S-MVPA	0.460	(0.383, 0.553)	<0.001
Alcohol consumption			
ABSTAINER	Reference		
LIGHT	0.676	(0.567, 0.805)	<0.001
MODERATE	0.593	(0.361, 0.944)	0.032
HEAVY	1.174	(0.714, 1.887)	0.517

CVD, cardiovascular diseases; HR, hazard ratio; MVPA, moderate to vigorous physical activity.

a Adjusted for age, gender, high-density lipoprotein (HDL), low-density lipoprotein (LDL), total cholesterol (TG), triglycerides (TC), body mass index (BMI), fasting plasma glucose (FPG), HbA1c and insulin.

### Joint effects of MVPA and alcohol use on all-cause mortality

Cox regression analyses were conducted to explore the association between the joint effect of physical activity with alcohol consumption and all-cause mortality (Table 4). There was a significantly lower risk of mortality among those in N-MVPA + LIGHT group (HR = 0.753; 95% CI = 0.585, 0.968; *p* = 0.027), I-MVPA + LIGHT group (HR = 0.400; 95% CI = 0.267, 0.588; *p* < 0.001), S-MVPA + ABSTAINER group (HR = 0.456; 95% CI = 0.355, 0.583; *p* < 0.001), S-MVPA + LIGHT group (HR = 0.358; 95% CI = 0.274, 0.456; *p* < 0.001), and S-MVPA + MODERATE group (HR = 0.348; 95% CI = 0.169, 0.667; *p* = 0.002) compared to the N-MVPA + ABSTRAINER group. The result indicated that S-MVPA + MODERATE group had the lowest CVD mortality compared with other groups (HR = 0.348, *p* = 0.002). For CVD patients with sufficient MVPA, light to moderate alcohol intake could provide better cardiovascular protection than those in ABSTAINER and HEAVY groups. While for CVD patients with insufficient MVPA, having light alcohol intake simultaneously could obtain greater protective effect of CVD compared with those only finish sufficient MVPA but never drink (HR = 0.400 vs. 0.456, *p* < 0.001). There was no statistically significant difference in all-cause mortality in N-MVPA + MODERATE group, N-MVPA + HEAVY group, L-MVPA + ABSTAINER group, I-MVPA + MODERATE group, I-MVPA + HEAVY group and S-MVPA + HEAVY group compared with N-MVPA + ABSTAINER group (*p* > 0.05). To eliminate the possibility that lower levels of physical activity and alcohol abstinence due to poor health conditions and multiple comorbidities, Student’s *t*-test was conducted and there was no significant difference between the N-MVPA + ABSTAINER and I-MVPA + LIGHT group (*p* = 0.229, Supplementary Table 1).

**Table 4:** Association between joint effect of physical activity with alcohol intake and all-cause mortality among the US adults with a history of CVD.

Groups	HR	95% CI	*p*-value
N-MVPA + ABSTAINER	Reference		
N-MVPA + LIGHT	0.753	(0.585, 0.968)	0.027
N-MVPA + MODERATE	0.529	(0.231, 1.133)	0.114
N-MVPA + HEAVY	1.265	(0.557, 2.731)	0.559
I-MVPA + ABSTAINER	0.776	(0.560, 1.072)	0.126
I-MVPA + LIGHT	0.400	(0.267, 0.588)	<0.001
I-MVPA + MODERATE	0.470	(0.124, 1.473)	0.219
I-MVPA + HEAVY	0.935	(0.187, 3.655)	0.928
S-MVPA + ABSTAINER	0.456	(0.355, 0.583)	<0.001
S-MVPA + LIGHT	0.358	(0.274, 0.466)	<0.001
S-MVPA + MODERATE	0.348	(0.169, 0.667)	0.002
S-MVPA + HEAVY	0.625	(0.314, 1.179)	0.161

CVD, cardiovascular diseases; HR, hazards ratio; MVPA, moderate to vigorous physical activity.

## Discussion

Our study provided a new insight into the relationship between joint physical activity and alcohol intake and all-cause mortality of CVD. The results showed that any level of exercise plus light to moderate alcohol use are both independently associated with reduced risk of all-cause mortality in CVD patients (graphical abstract).

In our analysis, among ethanol intake groups, both LIGHT and MODERATE were related to lower risk (HR = 0.676, *p* < 0.001 and HR = 0.593, *p* = 0.032, respectively) compared to ABSTAINER group. RCS analysis showed an inflection point in the dose-related curve, which means that drinking a small amount of alcohol (15.4 < drinks/week) might have cardiovascular protective effect, but as the amount of alcohol consumed increases, this protective effect quickly dissipates. For some specific diseases including malignant tumor (22) and liver diseases (18), there is no safe or beneficial level of alcohol consumption. For CVD, the benefits of moderate drinking were well documented. For example, a meta-analysis by Wood et al. (13) using data from 83 observational studies found that any alcohol consumption between 1.78 to 21 drinks/week was associated with a lower risk of MI incidence. Another meta-analysis indicated drinking ≤2 drinks/day was associated with decreased risk of ischemic stroke, whereas drinking over 2 drinks/day was associated with an increased risk of all stroke types (23), which is consistent with our results.

Two types of accounts could be proposed for this result. The first account assumes that CVD patients enhanced their cardiopulmonary function through exercise. Among MVPA categories, both I-MVPA and S-MVPA were associated with a significantly lower risk compared to N-MVPA group. These results demonstrated that in CVD patients, even exercise lower than the guidelines’ recommendation, still provides substantial cardiovascular protective effect compared to those who are physically inactive. The clinical benefits of exercise on cardiovascular health and physiological pathways linking to improved longevity in CVD patients are well documented (24–26). Exercise training has been prescribed as medicine for different CVD (27). Our findings are in accord with recent study that physically active participants had lower blood pressure, BMI, TG, TC, FPG, glycosylated hemoglobin, insulin level and higher HDL, revealing that the cardiovascular protective effects of exercise were related to the risk of cardiac metabolism reduction (24, 28). Studies have shown that exercise is beneficial to cardiovascular health through the IGF1/PI3K/AKT signaling pathway, indicating short-term appropriate AKT activation is beneficial to cardiovascular health, while long-term AKT activation or AKT overexpression is harmful to cardiovascular health (24). Our RCS analysis showed that continuing to increase the amount of exercise was still protective but brought less benefits, a possible explanation might be the heavy exercise does over-activated AKT protein kinase B, which resulted in the reduction of cardiovascular protective effect of physical activity.

The second account assumes that mechanisms proposed to explain the beneficial effects of moderate alcohol on cardiovascular variables include an increase in HDL. Our research found that as the amount of alcohol consumed increased, the HDL level of the participants also increased. HDL is an important factor for maintaining appropriate concentrations of LDL in vascular and other cells throughout the body. Additionally, HDL reduces adhesion molecule expression, inhibits oxidation of LDL, reduces thrombosis, and inhibits migration of inflammatory cells into the endothelial space. Acting principally through apolipoprotein A1, HDL also may have a direct antioxidant effect (29).

When taking both MVPA and alcohol use into consideration, CVD patients with I-MVPA keeping light alcohol intake, the relative risk of all-cause death decreased by 60%. CVD patients with S-MVPA and moderate ethanol consumption demonstrated the lowest risk of all-cause mortality, and the mortality risk among patients with S-MVPA and light alcoholic behavior was not significantly different compared to patients with S-MVPA and moderate alcohol use. An appropriate explanation could be the amount of alcohol intake and exercise play complementary roles in metabolic alterations in patients with CVD.

Firstly, among current drinkers, the observed correlation between ethanol consumption and mortality reduction may co-occur with improvement of insulin resistance (30) and type 2 diabetes (T2D), and physical activity also has a similar effect (31, 32). Other research had proposed that insulin resistance can damage endothelial function and alter the balance between vasoconstrictor and vasodilator mechanisms by numerous synergistic alterations (33, 34), which had adverse effect on cardiovascular system. Simultaneously, insulin resistance often accompanies the development of T2D, which exerts multifaceted and detrimental effects on vascular system, including endothelial dysfunction, arterial stiffness increases and accelerated atherosclerosis (35). Endothelial dysfunction, as a hallmark of T2D and insulin resistance, was characterized by impaired nitric oxide bioavailability, increased oxidative stress, and inflammatory responses within the vascular endothelium (36). This dysfunction contributes to increased arterial stiffness. Meanwhile, pathological changes of arteries can be further exacerbated by hyperglycemia induced cross-linking of collagen and other extracellular matrix components (35). This pathological mechanism reduces vascular compliance and is generally associated with a higher risk of CVD. We found that participants with higher alcohol consumption and MVPA had lower FPG, HbAlc and insulin. A meta-analysis of 20 cohort studies comprising 477,200 subjects confirmed the U-shaped relationship between moderate amount of alcohol consumption and risk of incident T2D (37). The amount of alcohol intake with better protective effect was 22 g/day for male and 24 g/day for female (37). According to the classification criteria in our study, 22 g/day or 24 g/day ethanol intake were equivalent to 11 drink/week and 12 drink/week alcohol intake, the same amount as the MODERATE group. A meta-analysis of prospective cohort studies by Crippa et al. (38) suggested that accumulating an activity volume which is commensurate with adherence to the current public health recommendations of 150 min of MVPA per week compared with sedentary individuals was associated with a reduction in the risk of T2D by 26% (95% CI 20, 31%) in the general population. Overall, we presumed that the joint moderate alcohol intake and physical activity reduce the incidence of T2D by improving insulin resistance, and indirectly lower the mortality rate of CVD.

Secondly, several studies have proved that alcohol consumption is associated with increased HDL levels (39–41), as well as exercise (42, 43), which can transport peripheral cholesterol to the liver thereby reducing lipid levels. The samples with light to moderate alcohol intake and MVPA had higher HDL level in our study. Some researchers had proposed the atherogenic index of plasma (AIP), which calculated using the formula log (TG/HDL), as a marker of plasma atherogenicity (44). Relevant research had indicated that higher AIP was significantly related to higher risk of developing major adverse cardiovascular events (MACEs) (45–47). The altered lipid profile usually caused by insulin resistance, which typically includes increase concentration of very-low-density lipoprotein (VLDL), HDL, formation of small dense LDL (sdLDL) and hypertriglyceridemia (48). The dyslipidemia can accelerate the formation of atherosclerotic plaques, which narrow the arterial lumen, restrict blood flow and finally lead to serious cardiovascular events such as myocardial infarction and stroke (49). Thus, we presumed that the beneficial effect of CVD from joint light to moderate ethanol consumption and MVPA was associated with AIP reducing.

Thirdly, alcohol consumption may transiently inhibit liver glucose output during exercise (50) to blunt the uptake of gluconeogenic precursors, resulting in an impaired hepatic gluconeogenesis (51). It reduces the amount of non-sugar substances converted into plasma glucose (50). Meanwhile, exercise training induces significant metabolic changes in the heart. Intense and prolonged exercise requires elevated carbohydrate oxidation within skeletal muscle with increased hepatic glycogenolysis and gluconeogenesis supplying the additional required glucose (52). Ultimately, exercise increases glucose utilization and alcohol intake decreases glucose production by the liver, thereby lowering FPG levels. Previously, alcohol ingestion prior to completing a 60-min cycling time trial in trained endurance cyclists decreased FPG concentrations (53). Furthermore, just as appropriate ethanol consumption can increase insulin sensitivity (54), physical training can increase insulin sensitivity in the trained muscle and thus reduces plasma glucose. The mechanisms include increased postreceptor insulin signaling, increased glucose transporter protein 4 (GLUT4) mRNA and protein, increased glycogen synthase activity and hexokinase, low release and increased clearance of free fatty acids, and increased transport of glucose to the muscles due to a larger muscle capillary network and blood flow (27).

We also noticed that healthy user effect, a well-documented source of bias in lifestyle epidemiology (55), could bring bias to the association between light-to-moderate alcohol consumption and reduced mortality. This phenomenon posits that individuals who adhere to one health recommendation (e.g., regular physical activity) are more likely to exhibit other unmeasured healthy behaviors, which collectively improve outcomes independent of the specific exposure under study. To minimize the bias, as shown in Tables 1, 2, factors that influence daily behaviors such as occupation, annual income, sleep status, and education level were compared between groups. There was no statistical difference in the distribution of occupations among the participants, and the income level did not differ significantly between the N-MVPA and I-MVPA groups. Although participants with more physical habits did exhibit higher annual income, less sleeping trouble and high education level, the survival advantage associated with low alcohol consumption may not only reflect biological benefits of ethanol, but also residual confounding from systemic health behaviors. Overall, non-drinkers should not initiate alcohol use given established health risks; Clinical priorities should focus on physical activity promotion per WHO guidelines.

The present study adds important results that among patients with established CVD, engaging in any amount of MVPA was associated with reduced all-cause mortality, while the observed correlation with light ethanol intake differed in mortality patterns compared to those who only exercise or drink alcohol. CVD patients usually have less amount of exercise compared to normal individuals. Previous study demonstrated that heart failure patients were on average 16% less physically active than individuals without a prior diagnosis of heart failure (56). For CVD patients who did not meet the recommended amount of physical activity, our results described an association between lower mortality and light alcohol consumption (less than 8.4 drinks/week), though this association may reflect confounding by health behaviors rather than causal effects of ethanol. Of note, alcohol’s carcinogenic and hepatotoxic effects may offset any potential metabolic improvements, major health organizations (e.g., WHO, AHA) explicitly discourage alcohol initiation for health benefits. Therefore, our study provides novel observational data exclusively supporting MVPA optimization, which will inform future guidelines focused on exercise-based interventions to improve CVD prognosis.

### Limitations

Our study has several limitations. Firstly, the diagnosis of CVD was self-reported without certificate of diagnosis issued by professional doctor. Additionally, clinically relevant information was not available, such as severity of symptoms, functional classification, hospitalizations and types of treatment received, which may further introduce the residual and unmeasured confounding. Secondly, both MVPA and alcohol use were not objectively measured but rather reported by the patients at baseline. Thirdly, the follow-up duration of the NHANES is relatively short which may introduce the bias due to reverse causation. Finally, our study could not investigate the effects of MVPA and alcohol use on top of current guideline-directed medical therapy.

## Conclusion

Among patients with established CVD, any level of MVPA was associated with survival benefits. The greatest mortality reduction occurred with >150 min/week of MVPA alongside light to moderate alcohol consumption (<15.4 drinks/week). However, comparable survival benefits were observed with <150 min/week of MVPA when coupled with light alcohol intake. MVPA remains the cornerstone for mortality risk reduction in this population.

## Data Availability

The datasets presented in this study can be found in online repositories. The names of the repository/repositories and accession number(s) can be found at: https://wwwn.cdc.gov/nchs/nhanes/Default.aspx.

## Footnotes

^1^ http://www.R-project.org/

## Glossary

NHANES: National Health and Nutrition Examination Survey
CVD: Cardiovascular disease
MVPA: Moderate-to-vigorous physical activity
BMI: Body mass index
non-HDL: Non-high-density lipoprotein
WHO: World health organization
CHD: Coronary heart disease
LDL: Low density lipoprotein
TG: Triglyceride
TC: Total cholesterol
FPG: Fasting plasma glucose
HbA1c: Hemoglobin type A1C
SBP: Systolic blood pressure
DBP: Diastolic blood pressure
MPA: Moderate intensity physical activity
VPA: Vigorous intensity physical activity
SD: Standard deviation
CI: Confidence interval
RCS: Restrict cubic spline
HR: Hazards ratio
ADH: Anti-diuretic hormone
T2D: Type 2 diabetes
AIP: Atherogenic index of plasma
MACEs: Major adverse cardiovascular events
VLDL: Very-low-density lipoprotein
sdLDL: Small dense LDL
GLUT4: Glucose transporter protein 4

## References

[1] Roth GA Johnson C Abajobir A Abd-Allah F Abera SF Abyu G. Global, regional, and national burden of cardiovascular diseases for 10 causes, 1990 to 2015. J Am Coll Cardiol. (2017) 70:1–25. doi: 10.1016/j.jacc.2017.04.052, PMID: 28527533 PMC5491406

[2] Roth GA Mensah GA Johnson CO Addolorato G Ammirati E Baddour LM. Global burden of cardiovascular diseases and risk factors, 1990–2019: update from the GBD 2019 study. J Am Coll Cardiol. (2020) 76:2982–3021. doi: 10.1016/j.jacc.2020.11.010, PMID: 33309175 PMC7755038

[3] Mensah GA Fuster V Murray CJL Roth GA Global Burden of Cardiovascular Diseases and Risks Collaborators. Global burden of cardiovascular diseases and risks, 1990–2022. J Am Coll Cardiol. (2023) 82:2350–473. doi: 10.1016/j.jacc.2023.11.007, PMID: 38092509 PMC7615984

[4] Mensah GA Fuster V Roth GA. A heart-healthy and stroke-free world: using data to inform global action. J Am Coll Cardiol. (2023) 82:2343–9. doi: 10.1016/j.jacc.2023.11.003, PMID: 38092508

[5] Perry AS Dooley EE Master H Spartano NL Brittain EL Pettee GK. Physical activity over the lifecourse and cardiovascular disease. Circ Res. (2023) 132:1725–40. doi: 10.1161/CIRCRESAHA.123.322121, PMID: 37289900 PMC10254078

[6] Chong B Jayabaskaran J Jauhari SM Chia J le Roux CW Mehta A. The global syndemic of modifiable cardiovascular risk factors projected from 2025 to 2050. J Am Coll Cardiol. (2025) 86:165–77. doi: 10.1016/j.jacc.2025.04.061, PMID: 40669954 PMC12718409

[7] Qiu X Wu Q Zhang Y Zhu Y Yang M Tao L. Association between life's essential 8 and frailty status among cancer survivors in the United States: a cross-sectional analysis. BMC Public Health. (2024) 24:1287. doi: 10.1186/s12889-024-18741-1, PMID: 38730364 PMC11088179

[8] Mok A Khaw KT Luben R Wareham N Brage S. Physical activity trajectories and mortality: population based cohort study. BMJ. (2019) 365:l2323. doi: 10.1136/bmj.l2323 31243014 PMC6592407

[9] Tikkanen E Gustafsson S Ingelsson E. Associations of fitness, physical activity, strength, and genetic risk with cardiovascular disease: longitudinal analyses in the UK Biobank study. Circulation. (2018) 137:2583–91. doi: 10.1161/CIRCULATIONAHA.117.032432, PMID: 29632216 PMC5997501

[10] Mi MY Perry AS Krishnan V Nayor M. Epidemiology and cardiovascular benefits of physical activity and exercise. Circ Res. (2025) 137:120–38. doi: 10.1161/CIRCRESAHA.125.325526, PMID: 40608856 PMC12233137

[11] Piercy KL Troiano RP Ballard RM Carlson SA Fulton JE Galuska DA. The physical activity guidelines for Americans. JAMA. (2018) 320:2020–8. doi: 10.1001/jama.2018.14854, PMID: 30418471 PMC9582631

[12] Bell S Daskalopoulou M Rapsomaniki E George J Britton A Bobak M. Association between clinically recorded alcohol consumption and initial presentation of 12 cardiovascular diseases: population based cohort study using linked health records. BMJ. (2017) 356:j909. doi: 10.1136/bmj.j909, PMID: 28331015 PMC5594422

[13] Wood AM Kaptoge S Butterworth AS Willeit P Warnakula S Bolton T. Risk thresholds for alcohol consumption: combined analysis of individual-participant data for 599,912 current drinkers in 83 prospective studies. Lancet. (2018) 391:1513–23. doi: 10.1016/S0140-6736(18)30134-X, PMID: 29676281 PMC5899998

[14] Roerecke M. Alcohol’s impact on the cardiovascular system. Nutrients. (2021) 13:3419. doi: 10.3390/nu13103419, PMID: 34684419 PMC8540436

[15] Biddinger KJ Emdin CA Haas ME Wang M Hindy G Ellinor PT. Association of habitual alcohol intake with risk of cardiovascular disease. JAMA Netw Open. (2022) 5:e223849. doi: 10.1001/jamanetworkopen.2022.3849, PMID: 35333364 PMC8956974

[16] Russo AM. Alcohol intake and risk of stroke in atrial fibrillation: the lesser the better, but this is not enough. Eur Heart J. (2021) 42:4769–71. doi: 10.1093/eurheartj/ehab657, PMID: 34590689

[17] Lee SR Choi EK Jung JH Han KD Oh S Lip GYH. Lower risk of stroke after alcohol abstinence in patients with incident atrial fibrillation: a nationwide population-based cohort study. Eur Heart J. (2021) 42:4759–68. doi: 10.1093/eurheartj/ehab315, PMID: 34097040 PMC8651176

[18] Aberg F Byrne CD Pirola CJ Mannisto V Sookoian S. Alcohol consumption and metabolic syndrome: clinical and epidemiological impact on liver disease. J Hepatol. (2023) 78:191–206. doi: 10.1016/j.jhep.2022.08.030, PMID: 36063967

[19] Kim Y Canada JM Kenyon J Billingsley H Arena R Lavie CJ. Physical activity, sedentary behaviors and all-cause mortality in patients with heart failure: findings from the NHANES 2007–2014. PLoS One. (2022) 17:e0271238. doi: 10.1371/journal.pone.0271238, PMID: 35839246 PMC9286289

[20] National Institute on Alcohol Abuse and Alcoholism (NIAAA). (2011). Alcohol’s effects on health research-based information on drinking and its impact. Available online at: https://www.niaaa.nih.gov/alcohol-health/overview-alcohol-consumption/moderate-binge-drinking (Accessed February, 2025).

[21] Abel EL Kruger ML Friedl J. How do physicians define “light,” “moderate,” and “heavy” drinking? Alcohol Clin Exp Res. (1998) 22:979–84. doi: 10.1111/j.1530-0277.1998.tb03692.x, PMID: 9726266

[22] Jung KJ Lee K Song DS Baek JW Shin SY Jee SH. Alcohol consumption and cancer risk in South Korea and the UK: prospective cohort studies. Int J Epidemiol. (2025) 54:dyaf108. doi: 10.1093/ije/dyaf108, PMID: 40574483 PMC12202743

[23] Larsson SC Wallin A Wolk A Markus HS. Differing association of alcohol consumption with different stroke types: a systematic review and meta-analysis. BMC Med. (2016) 14:178. doi: 10.1186/s12916-016-0721-4, PMID: 27881167 PMC5121939

[24] Chen H Chen C Spanos M Li G Lu R Bei Y. Exercise training maintains cardiovascular health: signaling pathways involved and potential therapeutics. Signal Transduct Target Ther. (2022) 7:306. doi: 10.1038/s41392-022-01153-1, PMID: 36050310 PMC9437103

[25] Lear SA Hu W Rangarajan S Gasevic D Leong D Iqbal R. The effect of physical activity on mortality and cardiovascular disease in 130,000 people from 17 high-income, middle-income, and low-income countries: the PURE study. Lancet. (2017) 390:2643–54. doi: 10.1016/S0140-6736(17)31634-3, PMID: 28943267

[26] Bennett DA Du H Clarke R Guo Y Yang L Bian Z. Association of Physical activity with risk of major cardiovascular diseases in Chinese men and women. JAMA Cardiol. (2017) 2:1349–58. doi: 10.1001/jamacardio.2017.4069, PMID: 29117341 PMC5814992

[27] Pedersen BK Saltin B. Exercise as medicine—evidence for prescribing exercise as therapy in 26 different chronic diseases. Scand J Med Sci Sports. (2015) 25:1–72. doi: 10.1111/sms.12581, PMID: 26606383

[28] Jenkins GP Evenson KR Herring AH Hales D Stevens J. Cardiometabolic correlates of physical activity and sedentary patterns in U.S. Youth Med Sci Sports Exerc. (2017) 49:1826–33. doi: 10.1249/MSS.0000000000001310, PMID: 28538259 PMC5976486

[29] Lucas DL Brown RA Wassef M Giles TD. Alcohol and the cardiovascular system: research challenges and opportunities. J Am Coll Cardiol. (2005) 45:1916–24. doi: 10.1016/j.jacc.2005.02.075 15963387

[30] Krittanawong C Isath A Rosenson RS Khawaja M Wang Z Fogg SE. Alcohol consumption and cardiovascular health. Am J Med. (2022) 135:1213–1230.e3. doi: 10.1016/j.amjmed.2022.04.021, PMID: 35580715 PMC9529807

[31] Myers J Kokkinos P Nyelin E. Physical activity, cardiorespiratory fitness, and the metabolic syndrome. Nutrients. (2019) 11:1652. doi: 10.3390/nu11071652, PMID: 31331009 PMC6683051

[32] Mac Gregor KA Gallagher IJ Moran CN. Relationship between insulin sensitivity and menstrual cycle is modified by BMI, fitness, and physical activity in NHANES. J Clin Endocrinol Metab. (2021) 106:2979–90. doi: 10.1210/clinem/dgab415, PMID: 34111293 PMC8475204

[33] Zhou MS Schulman IH Zeng Q. Link between the renin-angiotensin system and insulin resistance: implications for cardiovascular disease. Vasc Med. (2012) 17:330–41. doi: 10.1177/1358863X12450094, PMID: 22814999

[34] Muniyappa R Sowers JR. Role of insulin resistance in endothelial dysfunction. Rev Endocr Metab Disord. (2013) 14:5–12. doi: 10.1007/s11154-012-9229-1, PMID: 23306778 PMC3594115

[35] Lacolley P Regnault V Laurent S. Mechanisms of arterial stiffening: from mechanotransduction to epigenetics. Arterioscler Thromb Vasc Biol. (2020) 40:1055–62. doi: 10.1161/ATVBAHA.119.313129, PMID: 32075419

[36] Caturano A D’Angelo M Mormone A Russo V Mollica MP Salvatore T. Oxidative stress in type 2 diabetes: impacts from pathogenesis to lifestyle modifications. Curr Issues Mol Biol. (2023) 45:6651–66. doi: 10.3390/cimb45080420, PMID: 37623239 PMC10453126

[37] Baliunas DO Taylor BJ Irving H Roerecke M Patra J Mohapatra S. Alcohol as a risk factor for type 2 diabetes: a systematic review and meta-analysis. Diabetes Care. (2009) 32:2123–32. doi: 10.2337/dc09-0227, PMID: 19875607 PMC2768203

[38] Smith AD Crippa A Woodcock J Brage S. Physical activity and incident type 2 diabetes mellitus: a systematic review and dose-response meta-analysis of prospective cohort studies. Diabetologia. (2016) 59:2527–45. doi: 10.1007/s00125-016-4079-0, PMID: 27747395 PMC6207340

[39] Marques-Vidal P Bochud M Paccaud F Waterworth D Bergmann S Preisig M. No interaction between alcohol consumption and HDL-related genes on HDL cholesterol levels. Atherosclerosis. (2010) 211:551–7. doi: 10.1016/j.atherosclerosis.2010.04.001, PMID: 20430392

[40] Park H Kim K. Association of alcohol consumption with lipid profile in hypertensive men. Alcohol Alcohol. (2012) 47:282–7. doi: 10.1093/alcalc/ags019, PMID: 22371847

[41] Brien SE Ronksley PE Turner BJ Mukamal KJ Ghali WA. Effect of alcohol consumption on biological markers associated with risk of coronary heart disease: systematic review and meta-analysis of interventional studies. BMJ. (2011) 342:d636. doi: 10.1136/bmj.d636, PMID: 21343206 PMC3043110

[42] Nishida Y Hachiya T Hara M Shimanoe C Tanaka K Sutoh Y. The interaction between ABCA1 polymorphism and physical activity on the HDL-cholesterol levels in a Japanese population. J Lipid Res. (2020) 61:86–94. doi: 10.1194/jlr.P091546, PMID: 31694877 PMC6939595

[43] Hershey MS Sotos-Prieto M Ruiz-Canela M Martinez-Gonzalez MA Cassidy A Moffatt S. Anthocyanin intake and physical activity: associations with the lipid profile of a US working population. Molecules. (2020) 25:4398. doi: 10.3390/molecules25194398, PMID: 32987892 PMC7582364

[44] Qin Z Zhou K Li Y Cheng W Wang Z Wang J. The atherogenic index of plasma plays an important role in predicting the prognosis of type 2 diabetic subjects undergoing percutaneous coronary intervention: results from an observational cohort study in China. Cardiovasc Diabetol. (2020) 19:23. doi: 10.1186/s12933-020-0989-8, PMID: 32085772 PMC7035714

[45] Kim SH Cho YK Kim YJ Jung CH Lee WJ Park JY. Association of the atherogenic index of plasma with cardiovascular risk beyond the traditional risk factors: a nationwide population-based cohort study. Cardiovasc Diabetol. (2022) 21:81. doi: 10.1186/s12933-022-01522-8, PMID: 35599307 PMC9124430

[46] Fu L Zhou Y Sun J Zhu Z Xing Z Zhou S. Atherogenic index of plasma is associated with major adverse cardiovascular events in patients with type 2 diabetes mellitus. Cardiovasc Diabetol. (2021) 20:201. doi: 10.1186/s12933-021-01393-5, PMID: 34610830 PMC8493717

[47] Yao H Feng G Liu Y Chen Y Shao C Wang Z. Coronary artery calcification burden, atherogenic index of plasma, and risk of adverse cardiovascular events in the general population: evidence from a mediation analysis. Lipids Health Dis. (2024) 23:258. doi: 10.1186/s12944-024-02255-1, PMID: 39164730 PMC11334389

[48] Bjornstad P Eckel RH. Pathogenesis of lipid disorders in insulin resistance: a brief review. Curr Diab Rep. (2018) 18:127. doi: 10.1007/s11892-018-1101-6, PMID: 30328521 PMC6428207

[49] Hou P Fang J Liu Z Shi Y Agostini M Bernassola F. Macrophage polarization and metabolism in atherosclerosis. Cell Death Dis. (2023) 14:691. doi: 10.1038/s41419-023-06206-z, PMID: 37863894 PMC10589261

[50] Steiner JL Crowell KT Lang CH. Impact of alcohol on glycemic control and insulin action. Biomolecules. (2015) 5:2223–46. doi: 10.3390/biom5042223, PMID: 26426068 PMC4693236

[51] Suter PM Schutz Y. The effect of exercise, alcohol or both combined on health and physical performance. Int J Obes. (2008) 32:S48–52. doi: 10.1038/ijo.2008.206, PMID: 19079280

[52] Trefts E Williams AS Wasserman DH. Exercise and the regulation of hepatic metabolism. Prog Mol Biol Transl Sci. (2015) 135:203–25. doi: 10.1016/bs.pmbts.2015.07.010, PMID: 26477916 PMC4826571

[53] Marley A Bakali M Simpson C. Effect of a moderate alcohol dose on physiological responses during rest and prolonged cycling. Alcohol Alcohol. (2024) 59:agad079. doi: 10.1093/alcalc/agad079, PMID: 37981293 PMC10794168

[54] O’Keefe JH Bybee KA Lavie CJ. Alcohol and cardiovascular health. J Am Coll Cardiol. (2007) 50:1009–14. doi: 10.1016/j.jacc.2007.04.089, PMID: 17825708

[55] Li K Emerman I Cook AJ Fireman BH Sundaram M Tseng HX. Using double negative controls to adjust for healthy user bias in a recombinant zoster vaccine safety study. Am J Epidemiol. (2024). doi: 10.1093/aje/kwae439, PMID: 39604009 PMC12104472

[56] O’Donnell J Smith-Byrne K Velardo C Conrad N Salimi-Khorshidi G Doherty A. Self-reported and objectively measured physical activity in people with and without chronic heart failure: UK Biobank analysis. Open Heart. (2020) 7:e001099. doi: 10.1136/openhrt-2019-001099, PMID: 32153787 PMC7046950

