# Supplementary material for: Association between joint physical activity and alcohol intake and all-cause mortality in patients with cardiovascular disease: findings from NHANES 2007–2018
Source: Front Nutr. 2025 Sep 2;12:1616561. doi: 10.3389/fnut.2025.1616561 (PMC12436399; doi:10.3389/fnut.2025.1616561)
Supplement: Supplementary file 1 [file Table_1.DOCX]

**Supplymentary materials**

**Association between joint physical activity and alcohol intake and all-cause mortality in patients with cardiovascular disease: findings from NHANES 2007-2018**

Shiyang Zhang^a^, Yeji Zhuo^a^, Chunyan Zhu^a^, Tong Zhou^a^, Zongtao Wang^a^, Jianyi Zheng^a^, Tudi Li^a^, Rong Chen^a^, Dong Lin^a^, Zhixin Xie^a^, Zhenyang Fu^a^, Zhihuan Zeng^a,*^, Kaitong Chen^a,*^

**Supplementary Table 1 Current health status of participants in different groups**

| Group | t-statistic | P value |
| --- | --- | --- |
| N-MVPA+ ABSTAINER | Reference | |
| N-MVPA + LIGHT | 0.38 | 0.702 |
| N-MVPA+MODERATE | 0.07 | 0.943 |
| N-MVPA + HEAVY | -0.88 | 0.388 |
| I-MVPA+ ABSTAINER | 1.68 | 0.094 |
| I-MVPA + LIGHT | 2.33 | 0.229 |
| I-MVPA + MODERATE | 4.07 | 0.032 |
| I-MVPA + HEAVY | -0.98 | 0.354 |
| S-MVPA + ABSTAINER | 4.98 | <0.001 |
| S-MVPA +LIGHT | 6.89 | <0.001 |
| S-MVPA+ MODERATE | 2.34 | 0.263 |
| S-MVPA + HEAVY | 1.68 | 0.099 |

MVPA: moderate to vigorous physical activity.

Data which assess the current health levels of participants on questionnaire survey data from NHANES were collected. In this survey, participants need to answer, “During the past 30 days, how many days did pain make it hard for your to do usual activities, such as self-care, work, or recreation?”. Student’s t-test were used to evaluate the difference between groups.
